# Plant Alkaloids Inhibit Membrane Fusion Mediated by Calcium and Fragments of MERS-CoV and SARS-CoV/SARS-CoV-2 Fusion Peptides

**DOI:** 10.3390/biomedicines9101434

**Published:** 2021-10-10

**Authors:** Egor V. Shekunov, Svetlana S. Efimova, Natalia M. Yudintceva, Anna A. Muryleva, Vladimir V. Zarubaev, Alexander V. Slita, Olga S. Ostroumova

**Affiliations:** 1 Institute of Cytology of Russian Academy of Sciences, Tikhoretsky 4, 194064 Saint Petersburg, Russia; e.shekunov@alumni.nsu.ru (E.V.S.); yudintceva@incras.ru (N.M.Y.); ostroumova@incras.ru (O.S.O.); 2 Saint-Petersburg Pasteur Institute of Epidemiology and Microbiology, Mira 14, 197101 Saint Petersburg, Russia; anna.murka09099@mail.ru (A.A.M.); zarubaev@gmail.com (V.V.Z.); a_slita@yahoo.com (A.V.S.)

**Keywords:** alkaloids, membrane fusion, viral fusion inhibitor, antiviral therapy, COVID-19

## Abstract

To rationalize the antiviral actions of plant alkaloids, the ability of 20 compounds to inhibit calcium-mediated fusion of lipid vesicles composed of phosphatidylglycerol and cholesterol was investigated using the calcein release assay and dynamic light scattering. Piperine, tabersonine, hordenine, lupinine, quinine, and 3-isobutyl-1-methylxanthine demonstrated the most potent effects (inhibition index greater than 50%). The introduction of phosphatidylcholine into the phosphatidylglycerol/cholesterol mixture led to significant changes in quinine, hordenine, and 3-isobutyl-1-methylxanthine efficiency. Comparison of the fusion inhibitory ability of the tested alkaloids, and the results of the measurements of alkaloid-induced alterations in the physical properties of model membranes indicated a potent relationship between a decrease in the cooperativity of the phase transition of lipids and the ability of alkaloids to prevent calcium-mediated vesicle fusion. In order to use this knowledge to combat the novel coronavirus pandemic, the ability of the most effective compounds to suppress membrane fusion induced by fragments of MERS-CoV and SARS-CoV/SARS-CoV-2 fusion peptides was studied using the calcein release assay and confocal fluorescence microscopy. Piperine was shown to inhibit vesicle fusion mediated by both coronavirus peptides. Moreover, piperine was shown to significantly reduce the titer of SARS-CoV2 progeny in vitro in *Vero* cells when used in non-toxic concentrations.

## 1. Introduction

More than a third of FDA-approved compounds are natural substances of plant origins or their derivatives [1]. Plant compounds play a major role in the pharmaceutical industry due to their safety and relative cheapness [2,3,4]. Alkaloids are a wide class of nitrogen-containing compounds with basic properties numbering more than 8000 representatives [5]. Alkaloids demonstrate multiple biological activities, including antiviral [6], antibacterial [7], and antifungal actions [8]. Alkaloids were shown to be effective against *Retroviridae* [9,10,11,12], *Togaviridae* [13], *Herpesviridae* [14,15], *Flaviviridae* [16,17,18,19], *Filoviridae* [20], and *Orthomyxoviridae* [21,22]. Due to the global epidemiological situation, the ability of alkaloids to effectively fight viruses of the *Coronaviridae* family, including SARS-CoV-2, is of particular interest [23,24,25,26,27]. The use of alkaloids against COVID-19 were recently explored in clinical practices [28]. The literature indicates that the mechanisms of the antiviral activities of alkaloids are pleiotropic. For example, suppression of HIV by alkaloids, fagaronine, nitidine, columbamine, berberine, palmatine, and coraline is mediated by the inhibition of reverse transcriptase [29], while antiviral activity against the hepatitis B virus is related to the reduction of the activity of the p38 MAPK protein [30]. The extensive antiviral activity of alkaloids might suggest a more general and fundamental mechanism of their activity. Many viruses (influenza virus, HIV, SARS-CoV-2, Ebola virus, Zika virus, etc.), against which alkaloids are effective, are enveloped, i.e., they contain a lipid membrane [31]. The fusion of viral and cell membranes is a necessary stage for the viral development cycle, and blocking this stage is an effective therapeutic strategy [32]. Currently, there are several works devoted to the inhibition of viral fusion through alkaloid–peptide interactions [33,34]. It was revealed that alkaloids can inhibit viral fusion through interactions with cellular factors [35] and viral proteins [17,18,25,26,36]. However, the contribution of the lipid matrix and its biophysical properties to the fusion remains underestimated [37,38]. Research devoted to the study of fusion inhibitors, targeting the physical properties of viral and cell membranes, is still not sufficient [39].

Model lipid bilayers are usually used to rationalize and better understand the membrane fusion process [40,41,42]. Namely, the use of systems with specified physical parameters provides an accurate interpretation of the results and make it possible to reveal the molecular basis of the studied processes [42,43,44].

The aim of this work was to determine whether the antiviral activity of alkaloids is related to the ability to inhibit the fusion of the viral and cell membranes. The ability of 20 structurally different alkaloids to inhibit the calcium-induced fusion of lipid vesicles was evaluated, and the dependence of the alkaloid inhibitory activity on the lipid composition of fusing liposomes was studied. In order to demonstrate the relationship between changes in the physical properties of the lipid matrix and the suppression of membrane fusion under the action of alkaloids, the alkaloid-induced changes in the transmembrane distribution of lateral pressure and electrostatic potential were also assessed. To show that the data obtained could have direct implications for clinical practices, in terms of using plant alkaloids as fusion inhibitors, the alkaloid ability to inhibit lipid vesicle fusion triggered by crucial fragments of MERS-CoV and SARS-CoV/SARS-CoV-2 fusion peptides was additionally tested. The most effective compound, piperine, was also evaluated for in vitro antiviral activity against SARS-CoV-2 for *Vero* cells.

## 2. Materials and Methods

### 2.1. Materials

All chemicals used were of reagent grade. Synthetic 1,2-dioleoyl-sn-glycero-3-phospho-(1′-rac-glycerol) (DOPG), 1,2-dioleoyl-sn-glycero-3-phosphocholine (DOPC), 1-palmitoyl-2-oleoyl-sn-glycero-3-phosphocholine (POPC), sphingomyelin (Brain, Porcine) (SM), 1,2-dipalmitoyl-sn-glycero-3-phospho-(1′-rac-glycerol) (DPPG), cholesterol (CHOL), and 1,2-dipalmitoyl-sn-glycero-3-phosphoethanolamine-N-(lissamine rhodamine B sulfonyl) (Rh-DPPE) were obtained from Avanti^®^ Polar Lipids. Nonactin, KCl, CaCl_2_, PEG-8000, calcein, Triton X-100, Sephadex G-50, HEPES, DMSO, phosphate buffer solution (PBS), sorbitol, and alkaloids, atropine, (−)-lupinine, (−)-cotinine, berberine chloride, quinine, melatonin, caffeine, 1,7-dimethylxanthine, 3,9-dimethylxanthine, theophylline, 3-isobutyl-1-methylxanthine, 7-(β-hydroxyethyl) theophylline, pentoxifylline, hordenine, (±)-synephrine, colchicine, capsaicin, dihydrocapsaicin, and tabersonine, were purchased from Sigma-Aldrich Company Ltd. (Gillingham, UK). The chemical structures of the tested alkaloids are presented in Figure S1 (Supplementary Materials).

DMEM and DMEM/F12 culture mediums, fetal bovine serum (FBS), trypsin solution, and Dulbecco’s phosphate buffer solution (DPBS) were purchased from Gibco^TM^ (Life Technologies, Paisley, Scotland).

The modeling fusion peptides, FP-SARS-CoV-2 (RSFIEDLLFNKVT) and FP-MERS-CoV (RSAIEDLLFDKVT), were synthesized by the IQ Chemical LLC (Saint-Petersburg, Russia). The purity of peptides was ≥98%.

### 2.2. Calcein Leakage Assay

The choice of membrane-forming lipids is related to the enrichment of the viral lipid envelope with negatively charged and neutral glycerophospholipids, CHOL, and SM [45,46,47]. Small unilamellar vesicles were prepared from the mixtures of DOPG/CHOL (80/20 mol%), DOPC/DOPG/CHOL (40/40/20 mol%), and POPC/SM/CHOL (60/20/20 mol%) by extrusion. The resulting liposome suspension contained 3 mM lipid. Lipid stock in chloroform was dried under a gentle stream of nitrogen. Dry lipid film was hydrated by a buffer (35 mM calcein, 10 mM HEPES, pH 7.4). The suspension was subjected to five freeze–thaw cycles and then passed through a 100 nm nuclepore polycarbonate membrane for 13 times. The calcein that was not entrapped in the vesicles was removed by gel filtration with a Sephadex G-50 column to replace the buffer outside the liposomes with calcein-free solution (0.15 M NaCl, 10 mM HEPES, pH 7.4). Calcein inside the vesicles fluorescence very poorly, because of strong self-quenching at millimolar concentrations, while the fluorescence of disengaged calcein in the surrounding media is due to liposome fusion [41].

Here, CaCl_2_ and PEG-8000 were used as widely recognized fusion inducers [48,49,50,51]. The results of adding the different concentrations of CaCl_2_ into aqueous solutions, bathing calcein-loaded DOPG/CHOL (80/20 mol%) and DOPC/DOPG/CHOL (40/40/20 mol%) liposomes, are shown in Figure S2a,b (Supplementary Materials), respectively. Moreover, 20 and 40 mM CaCl_2_ was used to induce the fusion of DOPG/CHOL and DOPC/DOPG/CHOL liposomes, respectively.

The structure/function study performed by [49] showed that the fragment of MERS-CoV spike protein S2 subunit (RSARSAIEDLLFDKV), which is highly conserved throughout the coronavirus family, induces the dipalmitoylphosphatidylcholine/SM/CHOL liposome syncytium formation, and the IEDLLF core sequence has a key role in membrane perturbation. A shorter α-helical fragment of MERS-CoV fusion peptide (RSAIEDLLFDKVT) and the homologous highly conserved fragment of SARS-CoV/SARS-CoV-2 fusion peptides (RSFIEDLLFNKVT) [52] were chosen to mimic viral associated fusion. The chosen peptides were further referred to FP-MERS-CoV and FP-SARS-CoV-2, respectively. FP-SARS-CoV-2 and FP-MERS-CoV were added to POPC/SM/CHOL vesicles (60/20/20 mol%) to induce membrane fusion. The results of adding the different concentrations of peptides into aqueous solutions, bathing calcein-loaded POPC/SM/CHOL vesicles, are shown in Figure S2c,d (Supplementary Materials). Moreover, 50 µM FP-SARS-CoV-2 and 200 µM FP-MERS-CoV were used to induce liposome fusion to test the inhibitory action of alkaloids.

To test the fusion inhibitory action of alkaloids, a fusion inducer (calcium or modeling fusion peptides at the appropriate concentrations) was added to liposomes, pre-incubated with 400 μM of alkaloids (except for 40 μM of tabersonine). Since tabersonine at a concentration of 400 μM completely inhibited the calcium-mediated fusion of both DOPG/CHOL and DOPC/DOPG/CHOL vesicles, in order to understand whether its inhibitory effect depended on the lipid composition of liposomes, we reduced its concentration by 10-fold. Both concentrations of tabersonine (40 and 400 μM) were used to test its inhibitory action on the FP-SARS-CoV-2 and FP-MERS-CoV mediated fusion. 

We found that the osmotic pressure gradient resulting from the addition of a fusion inducer or an inhibitor did not affect the leakage of calcein from liposomes (data not shown).

The degree of calcein release was determined using a Fluorat-02-Panorama spectrofluorometer (Lumex, Saint-Petersburg, Russia). The excitation wavelength was 490 nm and the emission wavelength was 520 nm. Triton X-100 was added to a final concentration of 1% to each sample in order to completely disrupt liposomes, and the intensity after releasing the total amount of calcein from liposomes was measured. 

To describe the fusion of the liposomal membranes, the relative fluorescence of leaked calcein (*RF*, %) was calculated using the following formula:

(1) $$RF\;=\;\frac{I-I_{0}}{I_{\max}/0.9-I_{0}}\cdot 100\%,$$

where *I* and *I*_0_ were the calcein fluorescence intensities in the sample, in the presence and absence of the fusion inducer/inhibitor, respectively, and *I_max_* was the maximum fluorescence of the sample after lysis of liposomes by Triton X-100. Factor of 0.9 was introduced to calculate the dilution of the sample by Triton X-100.

The inhibition index (*II*) was calculated to characterize the relative efficiency of tested alkaloids to inhibit the fusion of liposomes compared to the action of the fusion inducer alone on the same liposome preparation:

(2) $$II=\;\frac{RF_{U\;}-RF_{S}}{RF_{U}}\cdot 100\%,$$

where *RF_U_* and *RF_S_* were the maximum relative fluorescence of the leaked calcein at the vesicle fusion induced by the calcium or modeling fusion peptides in the absence (*RF_U_*, *U = Ca^2+^/FP-MERS-CoV*/*FP-SARS-CoV-2*) and presence of alkaloids (*RF_S_*), respectively. The estimation of *RF_S_* was made, taking into account the intrinsic effect of alkaloids (the calcein leakage due to the potent lipid disordering action of alkaloid alone, *RF_A_*). Figure S3 and Table S1 (Supplementary Materials) present the kinetics and maximum values of *RF_A_* at the addition of different alkaloids into aqueous solutions, bathing calcein-loaded DOPG/CHOL (80/20 mol%) (Figure S3a), DOPC/DOPG/CHOL (40/40/20 mol%) (Figure S3b), and POPC/SM/CHOL (60/20/20 mol%) vesicles (Figure S3c). Time dependences of calcein leakage were fitted with two-exponential functions with characteristic times, *t*_1_ and *t*_2_, related to the fast and slow components of the release process.

### 2.3. Confocal Fluorescence Microscopy

The visualization of changes in the morphological features and behavior of vesicles in the suspension under the action of fusion inducers was performed by marking the liposome membranes by the fluorescent labeled lipid, Rh-DPPE. Giant unilamellar vesicles were prepared from the mixture of POPC/SM/CHOL (60/20/20 mol%) and 1 mol% of fluorescent lipid probe Rh-DPPE by the electroformation method (standard protocol, 3 V, 10 Hz, 1 h, 25 °C) using Nanion Vesicle Prep Pro (Munich, Germany). The resulting liposome suspension contained 1 mM lipid in 0.5 M sorbitol solution. The measurements of the liposome fusion were added; 10% of PEG-8000, 50 µM of FP-SARS-CoV-2, and 200 µM of MERS-CoV were used to induce membrane fusion and were incubated for 1–2 min at room temperature (25 ± 1 °C). Vesicles were imaged through an oil immersion objective (65 ×/1.4HCX PL) using an Olympus (Hamburg, Germany). A helium-neon laser with a wavelength of 561 nm was used to excite Rh-DPPE. The temperature during observation was controlled by air heating/cooling in a thermally insulated camera.

### 2.4. Dynamic Light Scattering

Small unilamellar vesicles composed of DOPG/CHOL (80/20 mol%) and POPC/SM/CHOL (60/20/20 mol%) were prepared by the extrusion and were treated with 400 μM of cotinine, melatonin, 3-isobutyl-1-methylxanthine, lupinine, piperine, hordenine, and 40 or 400 μM of tabersonine for 30 min before the addition of 20 mM CaCl_2_, 50 µM FP-SARS-CoV-2, and 200 µM MERS-CoV solution. Three different control samples were used: unmodified liposomes and vesicles incubated with fusion inducer, and the inhibitor alone. We found that the addition of alkaloid alone into the liposome suspension did not result in a noticeable alteration in vesicle size (data not shown). The hydrodynamic diameter (*d*, nm) and zeta-potential (ζ, mV) were determined on a Malvern Zetasizer Nano ZS 90 (Malvern Instruments Ltd., Malvern, United Kingdom) by gradual titration of liposome suspension in PBS at 25 °C. 

### 2.5. Membrane Boundary Potential Measurements

Virtually solvent-free planar lipid bilayers were prepared using a monolayer-opposition technique [53] on a 50-µm diameter aperture in a 10-µm thick Teflon film separating the two compartments of the Teflon chamber. The steady-state conductance of K^+^-nonactin was modulated via the two-sided addition of the colchicine, cotinine, 1,7-dimethylxanthine, capsaicin, synephrine, 3-isobutyl-1-methylxanthine, lupinine, piperine, tabersonine, and hordenine from different mM stock solutions in ethanol or methanol to the membrane-bathing solution (0.1 M KCl, pH 7.4) to obtain a final concentration ranging from 5 μM to 1 mM. The aperture was pretreated with hexadecane. Lipid bilayers were made from DOPG/CHOL (80/20 mol%). The conductance of the lipid bilayers was determined by measuring membrane conductance (*G*) at a constant transmembrane voltage (*V* = 50 mV). In the subsequent calculations, the membrane conductance was assumed to be related to the membrane boundary potential (*φ_b_*), the potential drop between the aqueous solution and the membrane hydrophobic core, by the Boltzmann distribution [54]:

(3) $$G\sim \xi \cdot C\exp(-\frac{ze\phi_{b}}{kT})\;$$

where *ξ* is the ion mobility, *ze* is the ion charge, *k* is the Boltzmann constant, and *T* is the absolute temperature.

Ag/AgCl electrodes with 1.5% agarose/2 M KCl bridges were used to apply *V* and measure *G*.

The current was measured using an Axopatch 200B amplifier (Molecular Devices, LLC, Orleans Drive, Sunnyvale, CA, USA) in the voltage clamp mode. Data were digitized using a Digidata 1440A and analyzed using pClamp 10.0 (Molecular Devices, LLC, Orleans Drive, Sunnyvale, CA, USA) and Origin 8.0 (OriginLab Corporation, Northampton, MA, USA). Data were acquired at a sampling frequency of 5 kHz using low-pass filtering at 200 Hz.

All experiments were performed at room temperature (25 °C).

### 2.6. Differential Scanning Microcalorimetry 

Differential scanning microcalorimetry experiments were performed by a μDSC 7EVO (Setaram, Caluire-et-Cuire, France). Giant unilamellar vesicles were prepared from a mixture of DPPG/CHOL (90/10 mol%) by the electroformation method (standard protocol, 3 V, 10 Hz, 1 h, 55 °C). The liposome suspension contained 3 mM lipid and was buffered by 5 mM HEPES at pH 7.4. The tested alkaloids (colchicine, cotinine, 1,7-dimethylxanthine, capsaicin, synephrine, 3-isobutyl-1-methylxanthine, lupinine, piperine, and hordenine) were added to aliquots to obtain a final concentration up to 400 μM of alkaloids (except for 40 μM of tabersonine). The liposomal suspension was heated at a constant rate of 0.2 C·min^−1^. The reversibility of the thermal transitions was assessed by reheating the sample immediately after the cooling step from the previous scan. The temperature dependence of the excess heat capacity was analyzed using Calisto Processing (Setaram, Caluire-et-Cuire, France). The peaks on the thermograms were characterized by the maximum temperatures of the main phase transition (*T_m_*) of DPPG/CHOL mixture and the half-width of the main peak (*T*_1/2_) indicating the size of the lipid cooperative unit. 

### 2.7. In Vitro Antiviral Activity Assessment 

To analyze the antiviral activity of piperine, monkey kidney epithelial cells, *Vero* (ATCC CCL81) were grown in culture vials (Nunc, Roskilde, Denmark) in DMEM culture medium supplemented with 10% FBS. The cell concentration was adjusted to 5 × 10^5^ cells/mL, and cells were seeded into 96-well culture plates (0.1 mL per well). The plates were incubated for 24 h at 37 °C in an atmosphere of 5% CO_2_. Piperine was dissolved in DMSO, and then two-fold serial dilutions of 200 to 1.56 μg/mL in serum-free DMEM/F12 nutrient medium were prepared. Afterwards, 100 μL/well of each dilution of piperine was added to *Vero* cells and incubated for 1 h at 37 °C in a 5% CO_2_ atmosphere. Serum-free DMEM/F12 medium was added to control wells instead of piperine. Aliquots of SARS-CoV-2 (10^3^ TCID50/mL) (isolate 17612) were mixed 1:1 with dilutions of piperine and incubated for 1 h at 37 °C. After 1 h incubation, piperine was removed from the plates, 100 μL/well of the SARS-CoV2/piperine mixture was added to the corresponding wells and incubated for 1 h at 37 °C in a 5% CO_2_ atmosphere, the virus-containing medium was added to the wells of the viral control, and serum-free DMEM/F12 was added to the cell control. The virus was then removed, the cells were washed three times with DPBS, 100 μL/well of dilutions of piperine (200–1.56 μg/mL) were added to the wells, serum-free DMEM/F12 medium was added to the control wells, and the plates were incubated for 24 h at 37 °C in a 5% CO_2_ atmosphere. After 24 h, supernatants from the experimental and control wells with the virus were collected and titrated by TCID_50_ assay in *Vero* cells after incubation for 72 h. The visual assessment of the cytopathogenic effect (CPE) of the virus was performed using an Olympus CKX41 light inverted microscope (Olympus, Tokyo, Japan).

### 2.8. Statistical Analysis

The control *RF_Ca2+_*-, *RF_FP-MERS-CoV_*-, *RF_FP-SARS-CoV-2_*-, *t*_1_-, and *t*_2_-values, characterizing the vesicle fusion induced by CaCl_2_, FP-SARS-CoV-2 or FP-MERS-CoV in the absence of alkaloids, were averaged from 5 to 9 independent experiments. The experimental *RF_S_*-, *t*_1_-, and *t*_2_-values, characterizing the CaCl_2_-, FP-SARS-CoV-2- and FP-MERS-CoV-mediated fusion of liposomes pretreated with different alkaloids, were averaged from 2 to 4 independent experiments. All *RF*-, *t*_1_-, and *t*_2_-values were presented as *mean* ± *standard error* of the mean (*p* ≤ 0.05). The magnitudes of *d, ζ*, *T_m_*, *T*_1/2_, and Δ*φ_b_* were averaged from 3 to 5 independent experiments and presented as *mean* ± *standard deviation* (*p* ≤ 0.05).

To prove the statistical significance of difference between *RF*-, *t*_1_-, *t*_2_-, *d*-, *ζ*-, *T_m_*-, and *T*_1/2_-values at the addition of alkaloids and the control values of related parameters (in the absence of alkaloids) the nonparametric signed-rank U-test (Mann–Whitney–Wilcoxon’s U-test) was employed (*—*p* ≤ 0.01, **—*p* ≤ 0.05).

Pearson’s correlation coefficient was applied to estimate the potent relationship between the inhibitory action of alkaloids on the vesicle fusion and the alkaloid-induced alterations in physical properties of lipid bilayers (Δ*T_m_*, Δ*T*_1/2_, Δ*φ_b_*). The coefficients were calculated using Microsoft Excel (Microsoft Corp., Redmond, WA, USA).

## 3. Results and Discussion

### 3.1. Effect of Alkaloids on CaCl_2_-Mediated Fusion of Negatively Charged Liposomes

Figure S4a–c (Supplementary Materials) shows the calcein leakage resulted from 20 mM CaCl_2_-mediated fusion of DOPG/CHOL-liposomes in the absence and presence of different alkaloids at 400 μM concentration (40 μM of tabersonine). Some alkaloids were able to inhibit calcium-mediated fusion, and this ability strictly depended on the alkaloid type (Figure S4b,c). Table 1 summarizes the mean values of maximum marker leakage caused by calcium-induced fusion of DOPG/CHOL vesicles pretreated by different alkaloids (*RF_S_*). The mean maximum leakage at the addition of fusion inducer alone is about 90%. A significant and reliable decrease in *RF_S_* (indicating the inhibition of calcium-mediated fusion) is caused by pretreatment of DOPG/CHOL vesicles with dihydrocapsaicin (*RF_S_* is about 70%), capsaicin, synephrine (*RF_S_* is about 50%), 3-isobutyl-1-methylxanthine, quinine, piperine (*RF_S_* is about 30%), tabersonine, hordenine (*RF_S_* is about 20%), and lupinine (*RF_S_* is about 10%). 

Table 1 also demonstrates the kinetic parameters of liposome fusion in the presence of alkaloids. It should be noted that the time dependences of the marker release due to vesicle fusion are well-described by two-exponential dependences with characteristic times related to fast and slow components (*t*_1_ and *t*_2_, respectively). Analysis of Table 1 indicates that *t*_1_ and *t*_2_ values vary in the ranges of about 1–10 and 10–120 min, respectively. The fast component might characterize the calcium sorption on the negatively charged DOPG-enriched membranes and the subsequent aggregation of the lipid vesicles, while the slow one might be related to the topological rearrangements of membrane lipids and further dye leakage at the liposome fusion. Colchicine, capsaicin, and tabersonine significantly accelerated the marker release kinetics at calcium-mediated fusion of DOPG/CHOL vesicles (about 5–10-fold decrease in both characteristic times is observed) (Table 1). Melatonin and quinine drastically slowed down the fusion process increasing *t*_2_ from about 70 to more than 100 min (Table 1).

**Figure 1:**
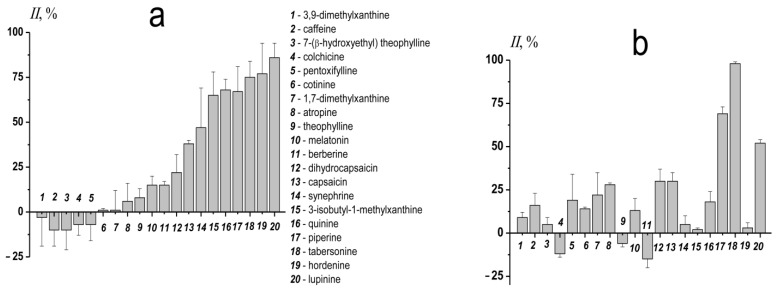
The inhibition index (*II*) characterizing the ability of tested alkaloids to suppress the fusion of vesicles made from DOPG/CHOL (80/20 mol%) (**a**) and DOPC/DOPG/CHOL (40/40/20 mol%) (**b**). Liposomes were incubated with 400 μM of alkaloids (except for 40 μM of tabersonine) for 30 min before the addition of CaCl_2_.

To characterize the relative efficiency of different alkaloids to inhibit calcium-mediated DOPG/CHOL liposome fusion the inhibition index (*II*) was calculated using Formula (2) for single preparation of liposome suspension, and then was averaged between the various preparations (Figure 1a). Taking into account the *II* value, the tested alkaloids were divided into three groups: ineffective (*II* does not exceed 15%); of low and medium efficiency (*II* is in the range of 16–50%), and of high efficiency (*II* is more than 50%). The first group includes 3,9-dimethylxanthine, caffeine, 7-(β-hydroxyethyl)theophylline, colchicine, pentoxifylline, cotinine, 1,7-dimethylxanthine, atropine, theophylline, melatonin, and berberine. The efficiency in the second group increases in the series: dihydrocapsaicin (*II* is about 25%) ≤ capsaicin ≈ synephrine (*II* is about 45%). The latter (the most effective) group includes 3-isobutyl-1-methylxanthine (*II* is about 65%), piperine ≈ quinine (*II* is about 70%), tabersonine (*II* is about 75%), hordenine (*II* is about 80%), and lupinine (*II* is about 85%).

**Figure 2:**
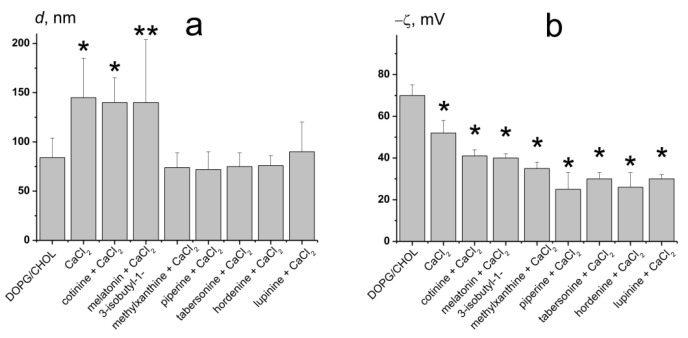
The diameter (*d*, nm) (**a**) and the ζ-potential (**b**) of the DOPG/CHOL (80/20 mol%) liposomes before and after addition of 20 mM CaCl_2_ to unmodified vesicles and liposomes pretreated by 400 μM of cotinine, melatonin, 3-isobutyl-1-methylxanthine, lupinine, piperine, hordenine, or 40 μM of tabersonine. *—*p* ≤ 0.01, **—*p* ≤ 0.05 (Mann–Whitney–Wilcoxon’s test, untreated liposomes vs. vesicles in the presence of CaCl_2_ or/and alkaloids).

Figure 2a shows the results of size measurements after calcium addition to unmodified DOPG/CHOL liposomes and vesicles pretreated by alkaloids of various anti-fusogenic activity, cotinine, melatonin, 3-isobutyl-1-methylxanthine, lupinine, piperine, tabersonine, and hordenine. The diameter of DOPG/CHOL liposomes in the absence of any modifiers is equal to 80 ± 20 nm. CaCl_2_ induces liposome fusion, and the vesicle size increased up to 145 ± 40 nm (Figure 2a). Cotinine and melatonin practically did not affect the size-increasing effect of calcium, as the liposome diameter was about 140 nm. The other tested compounds (lupinine, 3-isobutyl-1-methylxanthine, tabersonine, piperine, and hordenine) significantly inhibited calcium-mediated liposome fusion: the diameter of the vesicles was equal to 77 ± 13 nm (Figure 2a). The data obtained are in good agreement with the *II* values assessed by the calcein release assay (Figure 1a).

Figure 2b presents the results of estimation of the ζ-potential of DOPG/CHOL liposomes in the presence of 20 mM CaCl_2_ without and with different alkaloids. The ζ-potential of unmodified DOPG/CHOL liposomes was equal to about −70 mV. The addition of calcium led to an increase in ζ-potential up to about −50 mV. The ζ-potential value of DOPG/CHOL vesicles pretreated with alkaloids was 10–20 mV less than that of untreated ones (Figure 2b). The observed incomplete compensation of negative surface charge of DOPG-enriched liposomes was expected at the introduction of positively charged calcium ions and alkaloid molecules (lupinine, tabersonine, and hordenine). The disagreement of alkaloid abilities to inhibit calcium-mediated vesicle fusion and to increase ζ-potential (Figure 2a,b) clearly demonstrates that the anti-fusogenic activity of alkaloids is not due to partial compensation of the negative membrane surface charge by alkaloids or a competition between alkaloids and calcium ions for the interaction with negatively charged lipid groups. 

To study the dependence of the inhibition effect of alkaloids on the lipid composition of fusing vesicles, DOPC was introduced into membrane composition. It should be noted that the cell membranes are enriched of phosphatidylcholine species compared to virions [47,55,56]. The decrease in the proportion of negatively charged DOPG in membrane composition from 80 to 40 mol% was accompanied by an increase in CaCl_2_ concentration from 20 to 40 mM to reach the *RF_S_* value of about 80%. Figure S4d–f (Supplementary Materials) demonstrates the calcein leakage caused by the calcium-mediated fusion of DOPC/DOPG/CHOL liposomes. *RF_S_* resulted from calcium-mediated fusion of DOPC/DOPG/CHOL vesicles pretreated by different alkaloids reliably decreases in the series: caffeine ≈ pentoxifylline ≈ cotinine ≈ quinine (*RF_S_* is about 65%) ≥ atropine ≈ dihydrocapsaicin ≈ capsaicin (*RF_S_* is about 55%) > lupinine (*RF_S_* is about 40%) > piperine (*RF_S_* is about 25%) > tabersonine (*RF_S_* is equal to 1%) (Table 1).

The kinetic parameters of the time dependence of the calcein leakage caused by calcium-mediated fusion of DOPC/DOPG/CHOL liposomes are shown in Table 1. Colchicine and capsaicin accelerate the release kinetics due to calcium-mediated fusion of DOPC/DOPG/CHOL vesicles as well as of DOPG/CHOL liposomes. The huge inhibition of calcium-mediated fusion of DOPC/DOPG/CHOL liposomes in the presence of tabersonine did not allow for determining the characteristic times of dye release kinetics. Similar to DOPG/Chol vesicles, the pretreatment of DOPC/DOPG/CHOL liposomes with colchicine and capsaicin led to about five-fold decrease in *t*_2_-value. Unlike the case of DOPG/CHOL vesicles, the kinetics of calcein leakage due to fusion of DOPC/DOPG/CHOL liposomes was also accelerated by piperine (by six times), while theophylline and atropine slightly slowed down the fusion process, increasing *t*_2_ from 50 to about 100 and 120 min, respectively (Table 1).

Figure 1b shows the *II* values, characterizing the relative ability of different alkaloids to suppress the fusion of DOPC/DOPG/CHOL vesicles. Moreover, 3,9-dimethylxanthine, 7-(β-hydroxyethyl)theophylline, colchicine, 1,7-dimethylxanthine, theophylline, melatonin, berberine, synephrine, 3-isobutyl-1-methylxanthine, and hordenine did not demonstrate a significant ability to inhibit the fusion of DOPC/DOPG/CHOL liposomes. Caffeine, pentoxifylline, cotinine, atropine, dihydrocapsaicin, capsaicin, and quinine were characterized by low or medium efficiency to suppress the fusion of vesicles made of ternary lipid mixture, while the *II* values in the presence of lupinine, piperine, and tabersonine were about 50, 70, and 100% (Figure 1b). Comparison of Figure 1a,b clearly demonstrates that the anti-fusogenic activity of alkaloids strictly depends on the lipid composition of the vesicles.

### 3.2. The Influence of Alkaloids on the Physical Properties of the Model Lipid Membrane

It has been repeatedly shown that the elastic characteristics of the lipid bilayer play a huge role in the membrane fusion. The ordering of the lipid head groups and acyl chains, transbilayer lateral pressure profile, phase state of lipids, spontaneous curvature of the membrane, area per lipid molecule, and other characteristics can influence fusion [57]. Many of these parameters influence each other, but all of them are directly dependent on the lipid composition of the membrane. The key role of lipids is also supported by the fact that viral infections can alter cell lipid synthesis, regulating it according to their needs [58]. The ability of alkaloids and their derivatives to change the physical properties of a bilayer upon intercalation was demonstrated in a number of studies [59,60,61,62]. In particular, alkaloids affect the lipid packing [63,64], change lipid phase transition temperatures [65,66], etc. Recently, we performed a detailed study of alkaloid effects on the physical properties of the POPC membrane [67]. To understand the relationship between the alkaloid effects on the fusion and modulation of lipid matrix under their action, the influence of tested alkaloids on the properties of PG and CHOL-enriched bilayers were performed.

**Figure 3:**
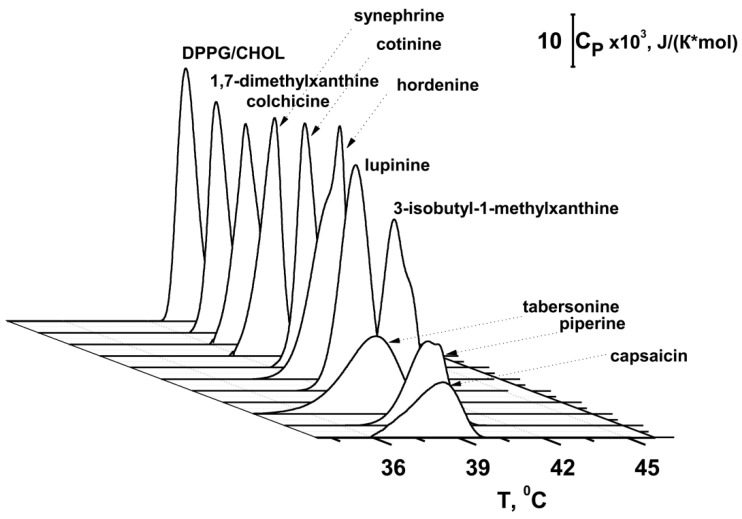
Heating thermograms of DPPG/CHOL (90/10 mol%) liposomes in the absence and presence of 400 μM of 1,7-dimethylxanthine, piperine, hordenine, cotinine, 3-isobutyl-1-methylxanthine, lupinine, synephrine, capsaicin, colchicine, and 40 μM of tabersonine.

Figure 3 presents the typical heating thermograms of DPPG/CHOL-liposomes in the absence (control) and in the presence of tested alkaloids. The value of the phase transition temperature (*T_m_*) of DPPG/CHOL mixture, and the half-width of the main peak (*T*_1/2_) are summarized in Table 2. *T_m_* is the point at which thermally induced lipid melting occurs [68], while *T*_1/2_ describes the sharpness of the phase transition or the inverse cooperativity of this process [69]. The temperature of the main transition (*T_m_*) of untreated DPPG/CHOL vesicles is equal to 40.4 °C, with a half-width of the peak (*T*_1/2_) of about 0.8 °C (Figure 3, control).

Colchicine, cotinine, and 1,7-dimethylxanthine did not affect the thermotropic phase behavior of DPPG/CHOL mixture (Figure 3, Table 2). Synephrine, 3-isobutyl-1-methylxanthine, lupinine, and hordenine decreased the *T_m_* by approximately 0.3 to 0.7 °C and increased the *T*_1/2_ by 0.4 to 0.6 °C (Figure 3, Table 2). The data are in agreement with the study of hordenine influence on the phase behavior of dimyristoylphosphatidylglycerol [61]. The introduction of capsaicin, piperine, and tabersonine was accompanied by a sharp decrease in *T_m_* (0.9–2.1 °C) and increase in *T*_1/2_ (0.7–1.2 °C) (Figure 3, Table 2). The correlation coefficients between Log*D_o/w_* (Table S1 Supplementary Materials) and –Δ*T_m_*- and Δ*T*_1/2_-values (Table 2) are in the range of 0.74–0.77, demonstrating a good correlation between the lipophilicity of the alkaloid molecules and their ability to alter lipid packing.

The correlation coefficient between the alkaloid-induced changes in *T_m_* of DPPG/CHOL (Table 2) and their *II* values in DOPG/CHOL vesicles (Figure 1a) was equal to 0.52, the corresponding coefficient characterizing the interdependence of alkaloid-induced changes in *T*_1/2_ (Table 2) and the *II* values (Table 1) was equal to 0.63. The correlation coefficient between the alkaloid-induced changes in *T_m_* (Table 2) and their *II* values in DOPC/DOPG/CHOL vesicles (Table 1) was equal to 0.64, while the interdependence of compound-induced changes in *T*_1/2_ (Table 2) and alkaloid *II* values of DOPC/DOPG/CHOL liposomes (Figure 1b) was characterized by a coefficient of 0.68. The observed correlation between the parameters characterizing the lipid melting, especially the cooperativity of lipid phase transition, and the fusion inhibition index indicates a relationship between the ability of alkaloids to disorder membrane lipids and to inhibit the liposome fusion. The incorporation of alkaloids into the polar region of the membrane, leading to an increase in lateral pressure, positive curvature stress, and an area per lipid molecule in this region, contributes to both a reduction in the temperature/cooperativity of the phase transition and an increase in the energy of fusion intermediates characterized by lipid surfaces of a negative curvature. Moreover, a comparison of the data obtained (Figure 3, Table 2) and our recently published results [65] demonstrate that the tested alkaloids have different effects on the phase behavior of DPPG/CHOL and DPPC. This fact might be related to the observed difference in their ability to inhibit the fusion of DOPG/CHOL and DOPC/DOPG/CHOL vesicles (Figure 1, Table 1). Alkaloid-induced curvature stress should depend on the depth of molecule insertion into lipid bilayer and its orientation in the membrane. The significant inhibitory action of 3-isobutyl-1-methylxanthine on the fusion of DOPG/CHOL liposomes might be related to the induction of the high positive curvature stress due to both the electrostatic interaction between xanthine residue and DOPG on the membrane surface and the tendency of the isobutyl side chain to be embedded into the membrane hydrocarbon core. Xanthine derivatives with lower hydrophobicity, i.e., caffeine, pentoxifylline, 1,7-dimethylxanthine, 3,9-dimethylxanthine, and 7-(β-hydroxyethyl)theophylline (Table S1, Supplementary Materials) did not inhibit calcium-mediated fusion of DOPG/CHOL liposomes. Introduction of DOPC into the membrane lipid composition was accompanied by a two-fold decrease in the DOPG content, which led to significant changes in the membrane orientation and embedment of xanthines. Thus, caffeine and pentoxifylline demonstrated a weak inhibitory effect on a fusion of DOPC/DOPG/CHOL vesicles, while 3-isobutyl-1-methylxanthine did not affect this process. The complete loss of the inhibiting ability of β-phenylethylamine derivatives, synephrine and hordenine, in membranes with lower content of DOPG compared to DOPG-enriched bilayers, could be explained in a similar way. An increase in the inhibitory effect of tabersonine on the fusion of DOPC/DOPG/CHOL vesicles compared to DOPG/CHOL liposomes might indicate its deeper immersion into the DOPC/DOPG/CHOL membranes than into DOPG/CHOL bilayers due to a decrease in the electrostatic interactions between positively charged tabersonine and negatively charged DOPG molecules.

Considering that liposome fusion is triggered by calcium ions, we also considered the possibility of alkaloids influencing fusion by changing the boundary potential of the membranes. Table 2 shows the maximum changes in *φ_b_* at the adsorption of different alkaloids (–Δ*φ_b_*(max)). The dependences of Δ*φ_b_* on the concentrations of tested alkaloids are presented in Figure S5 (Supplementary Materials). Tabersonine significantly increased the *φ_b_* of DOPG/CHOL membranes (by about 60 mV), while capsaicin dramatically decreased this value (by about −70 mV) (Figure S5 in the Supplementary Materials, Table 2). Colchicine, synephrine, 3-isobutyl-1-methylxanthine, lupinine, and piperine led to reduction in *φ_b_* by about 20–40 mV (Figure S5 in the Supplementary Materials, Table 2). Cotinine, 1,7-dimethylxanthine, and hordenine practically did not alter the *φ_b_*-value (Figure S5 Supplementary Materials, Table 2). There was no correlation between Δ*φ_b_* of DOPG/CHOL membranes after the addition of alkaloids and their *II* values. This fact demonstrates the minor role of changes in the bilayer electrical properties by alkaloids in their ability to inhibit the calcium-mediated membrane fusion.

### 3.3. Liposome Fusion Mediated by Fragments of Coronavirus Fusion Peptides

The COVID-19 pandemic has caused major challenges to healthcare systems across the globe. The lack of specific treatment, virus mutations, the emergence of new strains that have become resistant to vaccines, and many other factors, have led to the search for new drugs to treat COVID-19. To demonstrate the pharmacological applications of alkaloids as coronavirus fusion inhibitors—the abilities of the most effective compounds to suppress the membrane fusion induced by FP-MERS-CoV and FP-SARS-CoV-2 were studied. These peptides are able to induce calcein release due to fusion of POPC/SM/CHOL liposomes (Figure S2c,d Supplementary Materials), and are not effective to produce the fusion of DOPG/CHOL vesicles (data not shown).

**Figure 4:**
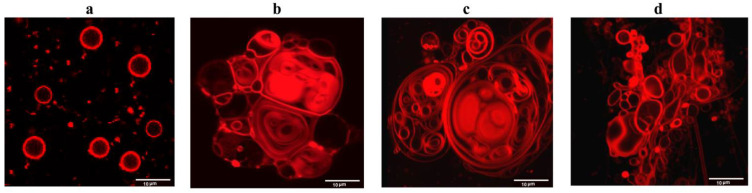
Fluorescence micrographs of giant unilamellar vesicles made from POPC/SM/CHOL (60/20/20 mol%) and 1 mol% of fluorescent lipid probe Rh-DPPE in the absence of any modifiers (**a**), and in the presence of 10% of PEG-8000 (**b**), 50 µM of FP-SARS-CoV-2 (**c**), and 200 µM of FP-MERS-CoV (**d**).

To further validate the fusogenic properties of FP-MERS-CoV and FP-SARS-CoV-2, the confocal fluorescence microscopy of giant unilamellar vesicles composed of POPC/SM/CHOL was performed. Figure 4 presents the fluorescence micrographs of POPC/SM/CHOL liposomes in the absence (Figure 4a) and presence of 10% of the well-established fusion inducer PEG-8000 (Figure 4b), 50 μM FP-SARS-CoV-2 (Figure 4c), and 200 μM FP-MERS-CoV (Figure 4d). Both peptides at the indicated concentrations caused deformation and aggregation of lipid vesicles, and increased in size. FP-SARS-CoV-2, unlike FP-MERS-CoV, also induced the formation of multilamellar and multivesicular liposomes (Figure 4c). In general, the morphological picture under the action of both peptides is similar to that which occurred when PEG-8000 was added (Figure 4b). Thus, the results obtained confirm the fusogenic activity of FP-SARS-CoV-2 and FP-MERS-CoV.

**Figure 5:**
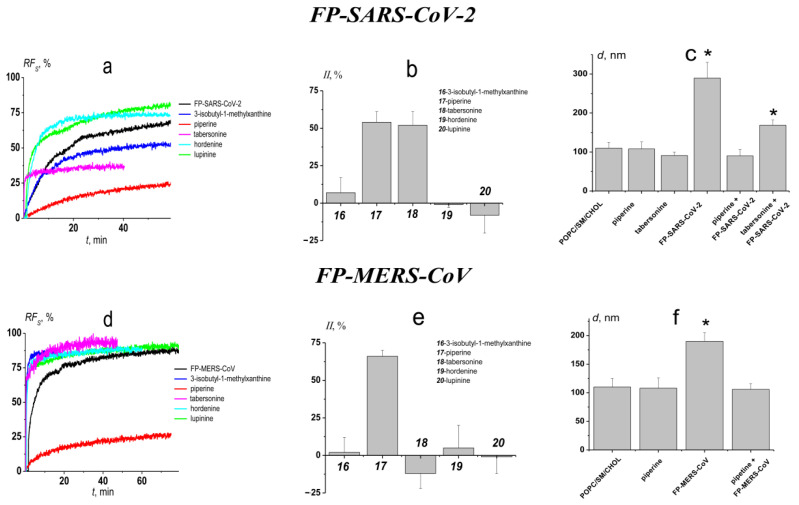
The effects of the alkaloids on FP-SARS-CoV-2 (upper panel) and FP-MERS-CoV (lower panel) mediated fusion of POPC/SM/CHOL (60/20/20 mol%) vesicles. (**a**,**d**) The time dependence of relative fluorescence of calcein (*RF_S_*, %) leaked due to the fusion induced by 50 μM of FP-SARS-CoV-2; (**a**) and 200 μM of FP-MERS-CoV; (**d**) in the absence and presence of alkaloids. Liposomes were incubated with 400 μM of alkaloids for 30 min before addition of the peptides. The relationship between the color line and the alkaloid is given in the figure. (**b**,**e**) The inhibition index (*II*) characterizing the ability of tested alkaloids to suppress the fusion induced by 50 μM of FP-SARS-CoV-2 (**b**); and 200 μM of FP-MERS-CoV (**e**). (**c**,**f**) The diameter (*d*, nm) of the POPC/SM/CHOL liposomes before and after addition of 50 μM of FP-SARS-CoV-2 (**c**) and 200 μM of FP-MERS-CoV (**f**) to vesicles pretreated with 400 μM of piperine or tabersonine. *—*p* ≤ 0.01 (Mann–Whitney–Wilcoxon’s test, untreated liposomes vs. vesicles in the presence of fusion peptides or/and alkaloids).

Figure 5a,d present the effects of alkaloids that effectively inhibit calcium-mediated fusion of DOPG/CHOL vesicles, 3-isobutyl-1-methylxanthine, piperine, tabersonine, hordenine, and lupinine, on the calcein leakage caused by FP-SARS-CoV-2- and FP-MERS-CoV-mediated fusion of POPC/SM/CHOL liposomes, respectively. In the absence of alkaloids *RF_s_*-value produced by 50 μM of FP-SARS-CoV-2 and 200 μM of FP-MERS-CoV was about 70 (Figure 5a) and 80% (Figure 5d), respectively. Table 3 summarizes the mean values of maximum marker leakage caused by peptide-induced fusion of POPC/SM/CHOL vesicles pretreated by different alkaloids and the kinetic parameters of liposome fusion in the presence of alkaloids. *RF_S_* caused by FP-SARS-CoV-2-mediated fusion of POPC/SM/CHOL vesicles significantly decreased in the presence of piperine (*RF_S_* is about 30%) and tabersonine (*RF_S_* is about 50%). Moreover, piperine was also effective against FP-MERS-CoV-mediated fusion (*RF_S_* is about 30%), while tabersonine was not characterized by an ability to inhibit FP-MERS-CoV-mediated fusion of POPC/SM/CHOL liposomes. The fusion-induced leakage in the presence of 3-isobutyl-1-methylxanthine, tabersonine, hordenine, and lupinine was characterized by faster kinetics compared to the absence of alkaloids, while highly active piperine slightly slowed down kinetics of FP-MERS-CoV-induced fusion by increasing *t*_2_-value. 

Figure 5b,e show the alkaloid *II*-values clearly demonstrating the ability of piperine to significantly inhibit both FP-SARS-CoV-2- and FP-MERS-CoV-mediated fusion of POPC/SM/CHOL liposomes, and the selectivity of tabersonine anti-fusogenic action. The observed selectivity of tabersonine action against liposome fusion induced by FP-SARS-CoV-2 and FP-MERS-CoV should be researched further.

To further demonstrate the anti-fusogenic activity of piperine, the vesicle size before and after the addition of FP-SARS-CoV-2 and FP-MERS-CoV to the unmodified and alkaloid pretreated POPC/SM/CHOL-liposomes was measured by dynamic light scattering (Figure 5c,f). In the absence of the any modifiers, the diameters of POPC/SM/CHOL-vesicles were equal to 110 ± 15 nm. Addition of the peptide FP-SARS-CoV-2 and FP-MERS-CoV led to an increased in size, up to 290 ± 40 nm and 190 ± 15 nm, respectively (Figure 5c,f). In the presence of piperine, the modeling fusion peptides were not able to increase the diameter of POPC/SM/CHOL-liposomes due to strong inhibition of vesicle fusion by the alkaloid. Figure 5c also demonstrates the ability of tabersonine to prevent the increase of liposome size under FP-SARS-CoV-2 action. The invariability of the liposome ζ-potential upon the addition of FP-SARS-CoV-2- and FP-MERS-CoV to both unmodified and alkaloid pretreated POPC/SM/CHOL liposomes is shown in Figure S6 (Supplementary Materials).

### 3.4. Antiviral Evaluation

Piperine is an alkaloid found in black pepper, one of the most widely used spices. The in vitro and in vivo antiviral activity of the alkaloid against the MERS-CoV in Vero cells, and in a mice model, was already demonstrated [70]. Here, we evaluated in vitro antiviral activity of piperine against the SARS-CoV-2 virus.

According to results of the cytotoxicity analysis performed by Hegeto et al. [71], the value of *IC*_50_ of piperine against *Vero* cells was equal to 183.33 µg/mL. Based on this finding, the maximum piperine concentration of 200 μg/mL was chosen. The incubation with 200 μg/mL of piperine for 72 h led to death of all cells in wells; all lower piperine concentrations used were nontoxic to *Vero* cells (data not shown).

To determine the infectious activity of viral progeny, ten-fold dilutions of the supernatants collected from the experimental wells and viral controls in serum-free DMEM/F12 medium were prepared. The resulting dilutions were added to a 96-well culture plate with 80–90% *Vero* cell monolayer and incubated for 72 h. After that, visual assessment of the cytopathogenic effect (CPE) of the virus was performed. The control samples without any additives demonstrated that the virus titer was 10^4^ TCID_50_/mL. Samples with a piperine concentration of 1.56 µg/mL resulted in 10^3^ TCID_50_/mL; alkaloid concentrations of 3.12–25 µg/mL had 10^2^ TCID_50_/mL, and in samples with piperine, concentrations of 50 and 100 µg/mL CPE were not observed at all. Thus, the significant reduction of the titer of SARS-CoV2 progeny in *Vero* cells at 1.56–100 µg/mL of piperine was clearly demonstrated.

## 4. Conclusions

New broad-spectrum antiviral drugs are needed due to the increase in the number of viral infections. Virus fusion inhibitors could be effective antiviral agents because fusion is a necessary step for the lifecycle of the virus. We used plant secondary metabolites, in particular, alkaloids, as a new class of fusion inhibitors. It was shown that the ability of alkaloids to inhibit calcium-mediated fusion of liposomes depends on their lipid composition. A correlation between the anti-fusogenic activity of alkaloids and their disordering effects on membrane lipids was found. Additionally, the ability of piperine to suppress the fusion induced by fragments of the coronavirus fusion peptides (MERS-CoV and SARS-CoV/SARS-CoV-2) was demonstrated. We also showed that piperine dramatically reduced the titer of SARS-CoV2 progeny in vitro in *Vero* cells when used in non-toxic concentrations. Thus, we hypothesize that the antiviral activity of piperine is related to its lipid-associated action. Moreover, to our knowledge, except for one bioassay with SARS-CoV-2-S pseudotyped particles [72], this is the first study demonstrating the in vitro activity of piperine against SARS-CoV-2 virus. It should be noted that piperine with curcumin is being studied in several clinical trials for the treatment of COVID-19 [73,74,75]. According to [75], administration of oral curcumin with piperine substantially reduced the morbidity and mortality due to COVID-19. We hope that, over time, the number of effective alkaloids in the treatment of COVID-19 will increase. The use of plant metabolites can successfully complete the existing therapeutic strategies, which will make it possible to combat the SARS-CoV-2 virus more effectively.

## Figures and Tables

**Table 1:** The parameters characterized the effects of alkaloids on the calcein release caused by the calcium-mediated fusion of liposomes composed of DOPG/CHOL (80/20 mol%) and DOPC/DOPG/CHOL (40/40/20 mol%).

	DOPG/CHOL			DOPC/DOPG/CHOL		
Alkaloid	*RF_S_*, %	*t*_1_, min	*t*_2_, min	*RF_S_*, %	*t*_1_, min	*t*_2_, min
*CaCl_2_*	92 ± 7	9 ± 2	68 ± 6	82 ± 1	5 ± 3	51 ± 12
*3,9-dimethylxanthine*	96 ± 2	2 ± 1 *	20 ± 1 *	69 ± 7	2 ± 1	82 ± 21
*caffeine*	94 ± 5	3 ± 2	38 ± 20	65 ± 8*	1 ± 1	94 ± 22
*7-(β-hydroxyethyl)theophylline*	94 ± 6	3 ± 1	36 ± 1 *	75 ± 1	1 ± 1	75 ± 31
*colchicine*	94 ± 4	1 ± 1 *	7 ± 1 *	87 ± 3	2 ± 1	14 ± 1 *
*pentoxifylline*	92 ± 6	2 ± 1 *	36 ± 13	64 ± 8 *	1 ± 1	60 ± 14
*cotinine*	87 ± 12	7 ± 2	46 ± 6 **	68 ± 2 *	3 ± 1	20 ± 9 *
*1,7-dimethylxanthine*	85 ± 3	8 ± 4	33 ± 5 *	60 ± 15	1 ± 1	64 ± 30
*atropine*	81 ± 6	7 ± 1	50 ± 9 **	52 ± 6 *	8 ± 1	119 ± 9 *
*theophylline*	79 ± 8	7 ± 3	61 ± 19	83 ± 2	11 ± 1	97 ± 7 *
*melatonin*	76 ± 9	10 ± 1	119 ± 24 *	84 ± 8	5 ± 3	51 ± 28
*berberine*	74 ± 11	2 ± 1 *	27 ± 6 *	92 ± 6	5 ± 2	33 ± 13
*dihydrocapsaicin*	67 ± 2 *	7 ± 1	48 ± 21	56 ± 4 *	2 ± 1	24 ± 11
*capsaicin*	53 ± 9 *	2 ± 1 *	14 ± 2 *	56 ± 2 *	3 ± 1	14 ± 5 *
*synephrine*	46 ± 20 *	5 ± 1	85 ± 12	77 ± 5	12 ± 4	64 ± 30
*3-isobutyl-1-methylxanthine*	32 ± 12 *	3 ± 1 *	68 ± 12	84 ± 4	9 ± 1	80 ± 24
*quinine*	27 ± 4 *	3 ± 1 **	118 ± 23 **	66 ± 3 *	3 ± 1	27 ± 10
*piperine*	27 ± 10 *	2 ± 1 *	27± 11 **	24 ± 1 *	2 ± 1	8 ± 2 *
*tabersonine*	21 ± 7 *	2 ± 1 *	11 ± 2 *	1 ± 1 *	– ^&^	– ^&^
*hordenine*	20 ± 16 *	1 ± 1 *	17 ± 6 *	82 ± 4	4 ± 3	50 ± 17
*lupinine*	13 ± 10 *	1 ± 1 *	90 ± 12	38 ± 4 *	6 ± 3	42 ± 3

*RF_S_*—the maximum leakage of fluorescent marker caused by the fusion of liposomes composed of DOPG/CHOL (80/20 mol%) and DOPC/DOPG/CHOL (40/40/20 mol%) mediated by 20 and 40 mM CaCl_2_ respectively. Liposomes have been incubated with 400 μM of alkaloids (except for 40 μM of tabersonine) for 30 min before the addition of CaCl_2_. *t*_1_ and *t*_2_—the time constants characterizing the fast (1) and slow (2) components of marker release kinetics (the time dependences of marker leakage were fitted by two-exponential functions). *—p ≤ 0.01, **—p ≤ 0.05 (Mann–Whitney–Wilcoxon’s test, CaCl_2_ alone vs. CaCl_2_ + alkaloid). ^&^—cannot be determined.

**Table 2:** The parameters characterized the effects of alkaloids on the physical properties of lipid bilayers: *T_m_*—the main transition temperature of DPPG/CHOL (90/10 mol%); *T*_1/2_—the half-width of the main peak in the presence of 400 μM of alkaloids (40 μM of tabersonine); Δ*φ_b_*(max)—the maximum changes in the boundary potential of DOPG/CHOL (80/20 mol%) membranes.

Alkaloid	*T_m_*, °C	*T*_1/2_, °C	Δ*φ_b_*(max), mV
*control*	40.4 ± 0.0	0.8 ± 0.0	–
*colchicine*	40.4 ± 0.0	0.8 ± 0.0	−24 ± 6
*cotinine*	40.4 ± 0.0	0.8 ± 0.0	−7 ± 3
*1,7-dimethylxanthine*	40.4 ± 0.0	0.8 ± 0.0	−5 ± 2
*capsaicin*	38.3 ± 0.2 *	2.0 ± 0.1 *	−70 ± 7
*synephrine*	40.1 ± 0.1 *	1.2 ± 0.1 *	−25 ± 3
*3-isobutyl-1-methylxanthine*	39.9 ± 0.2 *	1.2 ± 0.1 *	−18 ± 4
*piperine*	39.5 ± 0.3 *	1.5 ± 0.2 *	−39 ± 8
*tabersonine*	38.5 ± 0.2 *	1.9 ± 0.2 *	58 ± 9
*hordenine*	39.7 ± 0.2 *	1.2 ± 0.1 *	8 ± 3
*lupinine*	39.7 ± 0.1 *	1.5 ± 0.2 *	−30 ± 3

*—*p* ≤ 0.01 (Mann–Whitney–Wilcoxon’s test, control vs. alkaloid).

**Table 3:** The parameters characterized the effects of alkaloids on the calcein release caused by the FP-SARS-CoV-2 and FP-MERS-CoV-mediated fusion of liposomes composed of POPC/SM/CHOL.

	50 µM FP-SARS-CoV-2			200 µM FP-MERS-CoV		
Alkaloid	*RF_S_*, %	*t*_1_, min	*t*_2_, min	*RF_S_*, %	*t*_1_, min	*t*_2_, min
*inducer*	77 ± 6	11 ± 1	70 ± 4	80 ± 5	3 ± 2	22 ± 14
*3-isobutyl-1-methylxanthine*	66 ± 5	3 ± 1 *	15 ± 2 *	78 ± 6	2 ± 1	15± 5
*piperine*	33 ± 3 *	9 ± 3	73 ± 6	29 ± 2*	4 ± 1	48 ± 1 *
*tabersonine*	53 ± 9 **	1 ± 1*	27 ± 13 *	79 ± 7	2 ± 1	12 ± 2
*hordenine*	74 ± 8	3 ± 1*	18 ± 4 *	75 ± 10	2 ± 1	21 ± 1
*lupinine*	76 ± 4	1 ± 1*	31 ± 9 *	80 ± 6	1 ± 1	26 ± 1

*RF_S_*—the maximum leakage of fluorescent marker caused by the fusion of liposomes composed of POPC/SM/CHOL (60/20/20 mol%) mediated by 50 μM FP-SARS-CoV-2 or 200 μM FP-MERS-CoV. Liposomes have been incubated with 400 μM of alkaloids for 30 min before the addition of CaCl_2_. *t*_1_ and *t*_2_—the time constants characterizing the fast (1) and slow (2) components of marker release kinetics (the time dependences of marker leakage were fitted by two-exponential functions). *—*p* ≤ 0.01, **—*p* ≤ 0.05 (Mann–Whitney–Wilcoxon’s test, fusion peptide alone vs. fusion peptide + alkaloid).

## Supplementary Materials

The following are available online at https://www.mdpi.com/article/10.3390/biomedicines9101434/s1, Figure S1: The chemical structures of the tested alkaloids, Figure S2: The time dependence of relative fluorescence of calcein leaked from DOPG/CHOL (80/20 mol%) (a), DOPC/DOPG/CHOL (40/40/20 mol%) (b), and POPC/SM/CHOL (60/20/20 mol%) (c,d) vesicles induced by different concentrations of CaCl_2_ (a,b), FP-SARS-CoV-2 (c) and FP-MERS-CoV (d). The relationship between the color line and the concentration of the fusion inducers is given in the figure; Figure S3: The time dependence of relative fluorescence of calcein (*RF_A_*, %) leaked from DOPG/CHOL (80/20 mol%) (a), DOPC/DOPG/CHOL (40/40/20 mol%) (b), and POPC/SM/CHOL (60/20/20 mol%) (c) vesicles induced by alkaloids alone. Alkaloids were added into the liposomal suspension up to a concentration of 400 μM (except for 40 μM of tabersonine in DOPG/CHOL and DOPC/DOPG/CHOL vesicles) at the initial moment. The relationship between the color line and the compound is given in the figure; Figure S4. The time dependence of relative fluorescence of calcein (*RF_S_*, %) leaked by the fusion of DOPG/CHOL (80/20 mol%) (a–c) and DOPC/DOPG/CHOL (40/40/20 mol%) (d–f) vesicles induced by 20 mM of CaCl_2_ in the absence (*black line*) and presence of alkaloids. Liposomes were incubated with 400 μM of alkaloids (except for 40 μM of tabersonine) for 30 min before the addition of CaCl_2_. The relationship between the color line and the alkaloid is given in the figure; Figure S5: The dependence of the changes in the membrane boundary potential (Δ*φ_b_*) on the concentration of colchicine (*), cotinine (▲), 1,7-dimethylxanthine (◼), capsaicin (◯), synephrine (◊), 3-isobutyl-1-methylxanthine (●), lupinine (□), piperine (▼), tabersonine (♦), and hordenine (◄). The membranes were composed of DOPG/CHOL (80/20 mol%) and bathed in 0.1 M KCl at pH 7.4. *V* = 50 mV, Figure S6: The ζ-potential of the POPC/SM/CHOL (60/20/20 mol%) liposomes before and after addition of 50 µM of FP-SARS-CoV-2 or 200 µM of FP-MERS-CoV to unmodified and pretreated by 400 μM of piperine vesicles; Table S1: The parameters characterized the alkaloid molecules and their influence on the calcein leakage from liposomes of different composition.
