# Supplementary material for: Plant Alkaloids Inhibit Membrane Fusion Mediated by Calcium and Fragments of MERS-CoV and SARS-CoV/SARS-CoV-2 Fusion Peptides
Source: Biomedicines. 2021 Oct 10;9(10):1434. doi: 10.3390/biomedicines9101434 (PMC8533497; doi:10.3390/biomedicines9101434)
Supplement: Supplementary file 1 [file biomedicines-09-01434-s001.zip › biomedicines-1376060-supplementary.pdf]

Supplementary materials

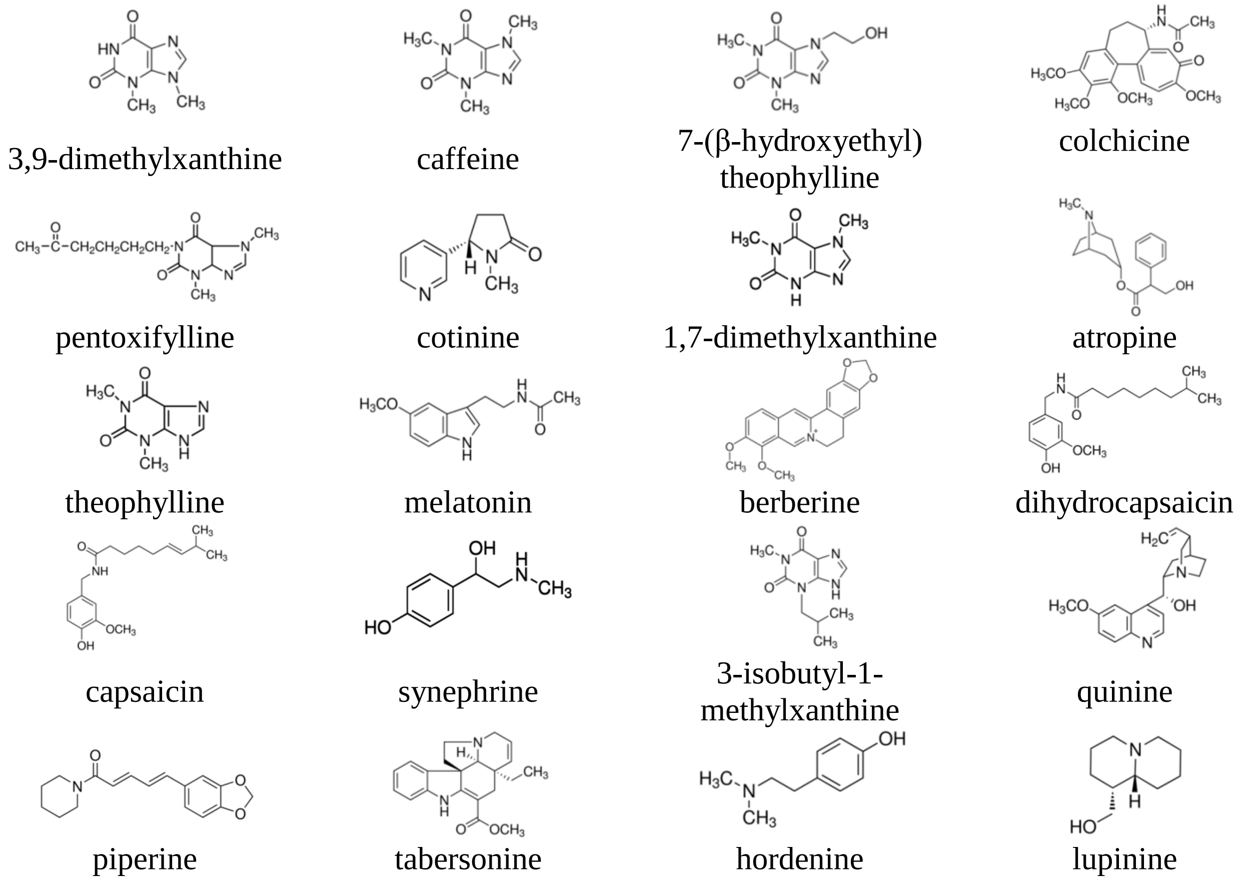

Figure S1. The chemical structures of the tested alkaloids.

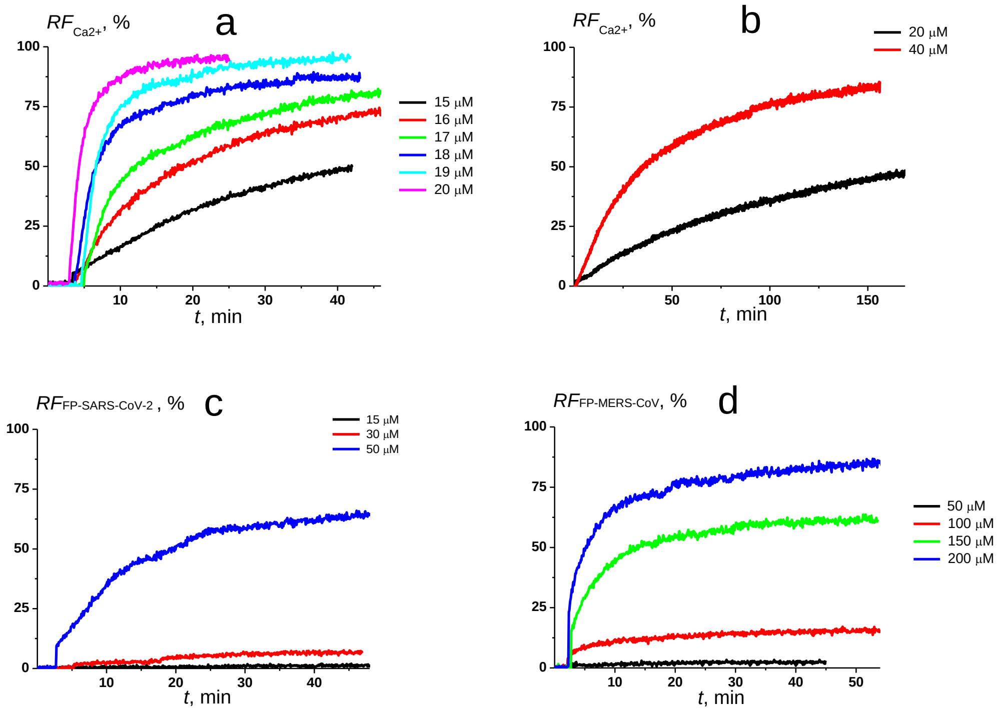

Figure S2. The time dependence of relative fluorescence of calcein leaked from DOPG/CHOL (80/20 mol%) (**a**), DOPC/DOPG/CHOL (40/40/20 mol%) (**b**), and POPC/SM/CHOL (60/20/20 mol%) (**c**, **d**) vesicles induced by different concentrations of $CaCl_2$ (**a**, **b**), FP-SARS-CoV-2 (**c**) and FP-MERS-CoV (**d**). The relationship between the color line and the concentration of the fusion inducers is given on the figure.

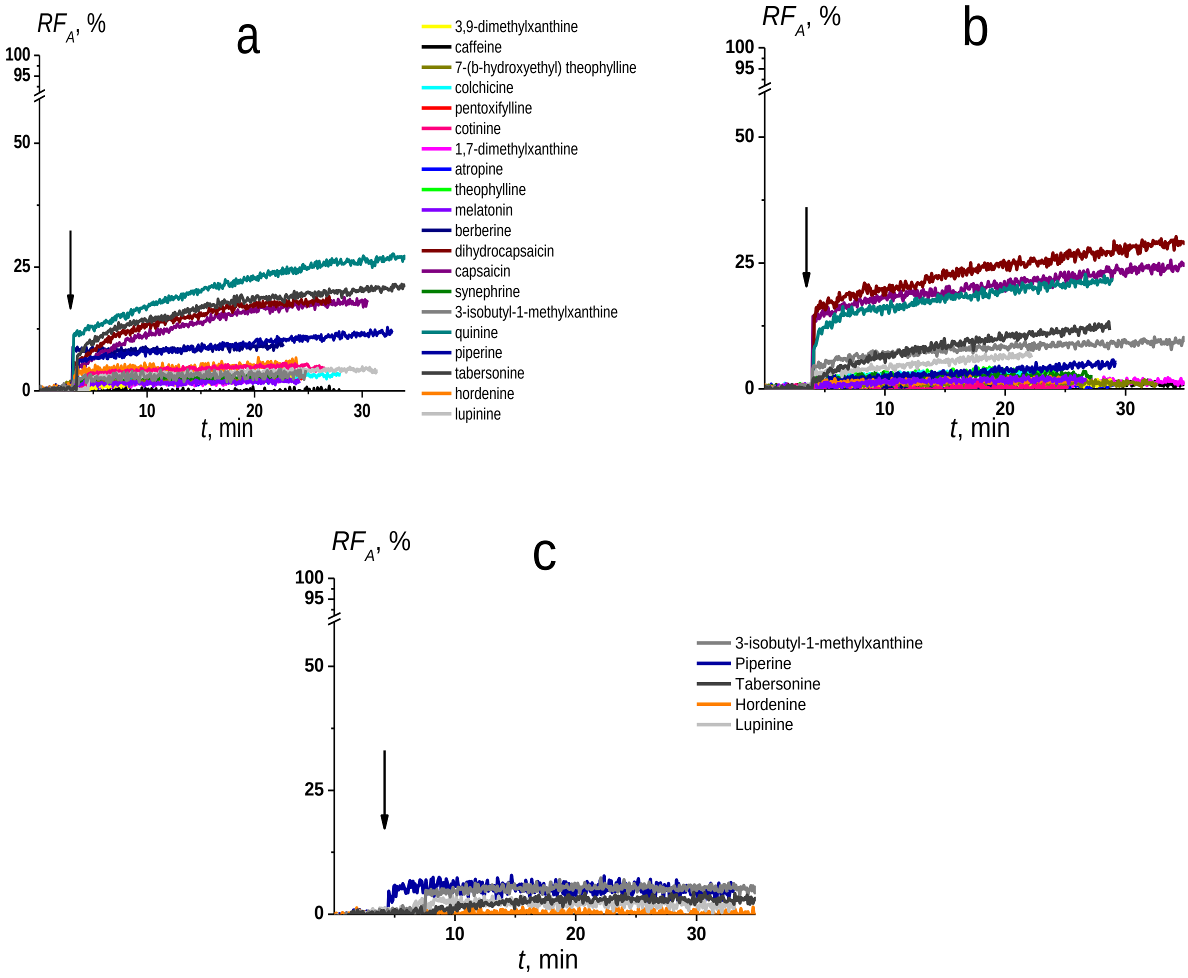

Figure S3. The time dependence of relative fluorescence of calcein (RF_A, %) leaked from DOPG/CHOL (80/20 mol%) (**a**), DOPC/DOPG/CHOL (40/40/20 mol%) (**b**), and POPC/SM/CHOL (60/20/20 mol%) (**c**) vesicles induced by alkaloids alone. Alkaloids were added into the liposomal suspension up to a concentration of 400 µM (except for 40 µM of tabersonine in DOPG/CHOL and DOPC/DOPG/CHOL vesicles) at the initial moment. The relationship between the color line and the compound is given on the figure.

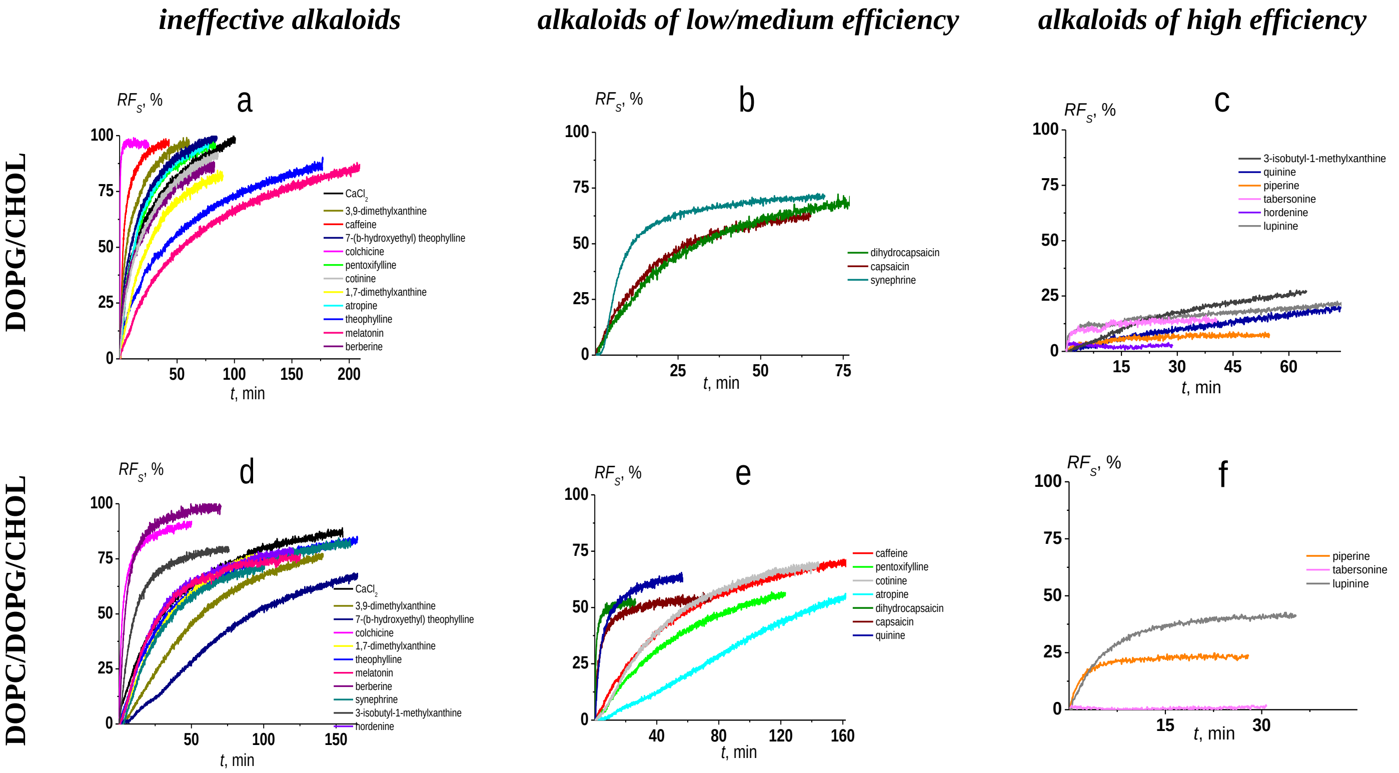

Figure S4. The time dependence of relative fluorescence of calcein (RF_S, %) leaked by the fusion of DOPG/CHOL (80/20 mol%) (**a**, **b**, **c**) and DOPC/DOPG/CHOL (40/40/20 mol%) (**d**, **e**, **f**) vesicles induced by 20 mM of $CaCl_2$ in the absence (*black line*) and presence of alkaloids. Liposomes were incubated with 400 µM of alkaloids (except for 40 µM of tabersonine) for 30 min before the addition of $CaCl_2$. The relationship between the color line and the alkaloid is given on the figure.

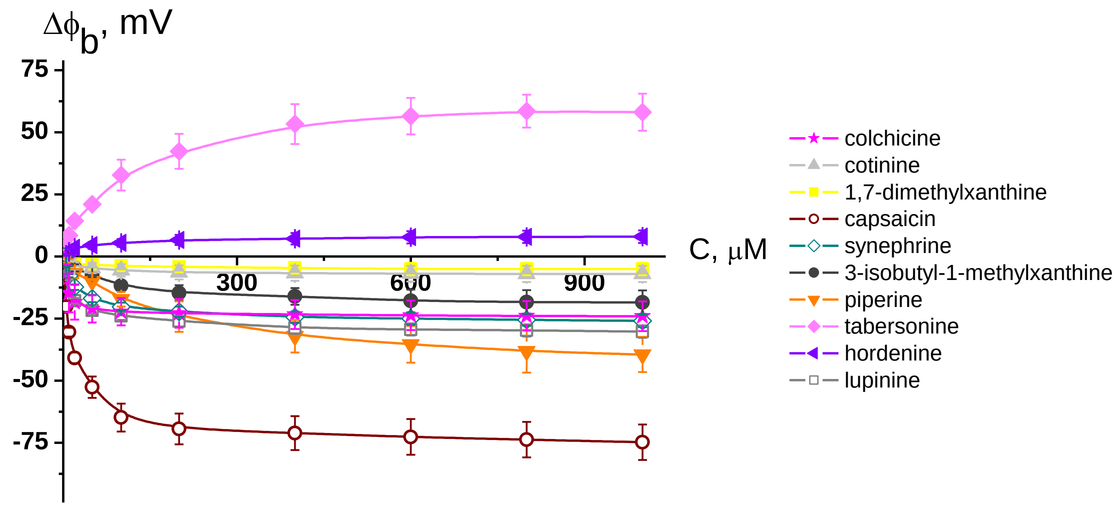

Figure S5. The dependence of the changes in the membrane boundary potential ($\Delta\varphi_b$) on the concentration of colchicine (*), cotinine (▲), 1,7-dimethylxanthine (■), capsaicin (○), synephrine (◊), 3-isobutyl-1-methylxanthine (●), lupinine (□), piperine (▼), tabersonine (♦), and hordenine (◄). The membranes were composed of DOPG/CHOL (80/20 mol%) and bathed in 0.1 M KCl at pH 7.4. V = 50 mV.

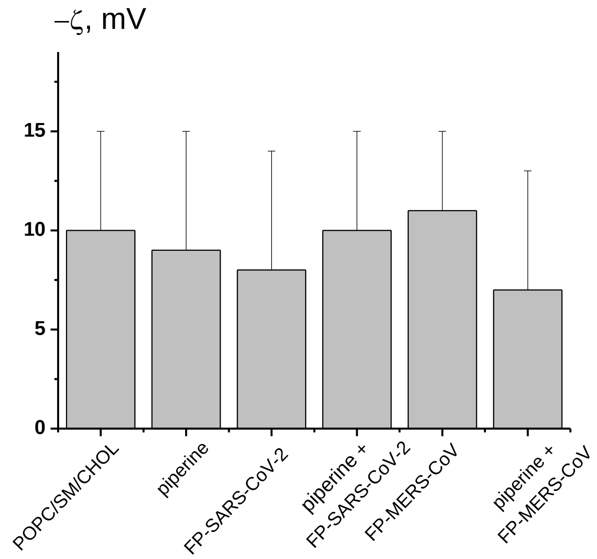

Figure S6. The ζ-potential of the POPC/SM/CHOL (60/20/20 mol%) liposomes before and after addition of 50 μM of FP-SARS-CoV-2 or 200 μM of FP-MERS-CoV to unmodified and pretreated by 400 μM of piperine vesicles.

Table S1. The parameters characterized the alkaloid molecules and their influence on the calcein leakage from liposomes of different composition.

alkaloid	***charge****	***$LogD_{o/w}$****	***RF_A, %***		
			DOPG/CHOL	***DOPC/DOPG/CHOL***	***POPC/SM/CHOL***
3,9-dimethylxanthine	-0.02	-0.82	4 ± 2	3 ± 2	–
caffeine	0	-0.55	3 ± 2	2 ± 1	–
7-(β-hydroxyethyl) theophylline	0	-1.24	4 ± 2	2 ± 1	–
colchicine	0	1.46	4 ± 2	4 ± 3	–
pentoxifylline	0	0.23	2 ± 1	2 ± 1	–
cotinine	0	0.21	6 ± 2	3 ± 2	–
1,7-dimethylxanthine	0	0.24	4 ± 2	2 ± 1	–
atropine	0.99	-0.41	2 ± 1	2 ± 1	–
theophylline	-0.28	-0.89	5 ± 2	4 ± 2	–
melatonin	0	1.15	3 ± 2	3 ± 2	–
berberine	1	-1.28	11 ± 4	5 ± 3	–
dihydrocapsaicin	0	4.11	20 ± 7	26 ± 6	–
capsaicin	0	3.75	19 ± 7	24 ± 5	–
synephrine	0.97	-1.39	5 ± 2	4 ± 2	–
3-isobutyl-1-methylxanthine	-0.09	0.4	7 ± 4	9 ± 5	8 ± 4
quinine	0.98	0.86	24 ± 8	20 ± 6	–
piperine	0	2.78	12 ± 4	8 ± 3	7 ± 4
tabersonine	0.98	0.9	19 ± 4$^{@}$	14 ± 3$^{@}$	5 ± 3
hordenine	0.98	0.06	8 ± 4	5 ± 2	2 ± 1
lupinine	1	-1.52	6 ± 3	7 ± 4	3 ± 2

* the values of the total electrical charge and the logarithm of octanol/water distribution coefficient at pH 7.4, $LogD_{o/w}$, were predicted by ChemAxon.

RF_A – the maximal leakage of the fluorescent marker from vesicles composed of DOPG/CHOL (80/20 mol%), DOPC/DOPG/CHOL (40/40/20 mol%), and POPC/SM/CHOL (60/20/20 mol%)) induced by 400 μM of alkaloids (@ – 40 μM of tabersonine was used to modify DOPG/CHOL and DOPC/DOPG/CHOL liposomes).
